# Dropout in cognitive behavioral treatment in adults living with overweight and obesity: a systematic review

**DOI:** 10.3389/fnut.2024.1250683

**Published:** 2024-05-09

**Authors:** Lenycia de Cassya Lopes Neri, Francesca Mariotti, Monica Guglielmetti, Simona Fiorini, Anna Tagliabue, Cinzia Ferraris

**Affiliations:** ^1^Human Nutrition and Eating Disorder Research Center, Department of Public Health, Experimental and Forensic Medicine, University of Pavia, Pavia, Italy; ^2^Laboratory of Food Education and Sport Nutrition, Department of Public Health, Experimental and Forensic Medicine, University of Pavia, Pavia, Italy

**Keywords:** dropout, predictive factors, cognitive behavioral therapy, cognitive behavioral treatment, nutritional counseling, attrition, overweight, systematic review

## Abstract

Obesity is a chronic, complex, and multifactorial disease resulting from the interaction of genetic, environmental, and behavioral factors. It is characterized by excessive fat accumulation in adipose tissue, which damages health and deteriorates the quality of life. Although dietary treatment can significantly improve health, high attrition is a common problem in weight loss interventions with serious consequences for weight loss management and frustration. The strategy used to improve compliance has been combining dietary prescriptions and recommendations for physical activity with cognitive behavioral treatment (CBT) for weight management. This systematic review determined the dropout rate and predictive factors associated with dropout from CBT for adults with overweight and obesity. The data from the 37 articles selected shows an overall dropout rate between 5 and 62%. The predictive factors associated with attrition can be distinguished by demographics (younger age, educational status, unemployed status, and ethnicity) and psychological variables (greater expected 1-year Body Mass Index loss, previous weight loss attempts, perceiving more stress with dieting, weight and shape concerns, body image dissatisfaction, higher stress, anxiety, and depression). Common reasons for dropping out were objective (i.e., long-term sickness, acute illness, and pregnancy), logistical, poor job conditions or job difficulties, low level of organization, dissatisfaction with the initial results, lack of motivation, and lack of adherence. According to the Mixed Methods Appraisal quality analysis, 13.5% of articles were classified as five stars, and none received the lowest quality grade (1 star). The majority of articles were classified as 4 stars (46%). At least 50% of the selected articles exhibited a high risk of bias. The domain characterized by a higher level of bias was that of randomization, with more than 60% of the articles having a high risk of bias. The high risk of bias in these articles can probably depend on the type of study design, which, in most cases, was observational and non-randomized. These findings demonstrate that CBT could be a promising approach for obesity treatment, achieving, in most cases, lower dropout rates than other non-behavioral interventions. However, more studies should be conducted to compare obesity treatment strategies, as there is heterogeneity in the dropout assessment and the population studied. Ultimately, gaining a deeper understanding of the comparative effectiveness of these treatment strategies is of great value to patients, clinicians, and healthcare policymakers.

**Systematic review registration**: PROSPERO 2022 CRD42022369995 Available from: https://www.crd.york.ac.uk/prospero/display_record.php?ID=CRD42022369995.

## Introduction

It is well known that obesity is a significant public health burden (1), affecting both physical and psychological status. According to the recently published evidence-based practice guide of the Academy of Nutrition and Dietetics, obesity is recognized as excess adiposity. It is correlated with many adverse health outcomes, such as mortality risk, prediabetes, type 2 Diabetes Mellitus (T2DM), cardiovascular disease, obstructive sleep apnea, and certain types of cancer (2, 3). Dietary administration combined with physical activity is the most recommended treatment for weight loss (4).

Although dietary treatment can significantly improve health, dietary modifications are difficult to make on an individual basis, and obstacles to changing behavior may also be influenced by psychological factors in addition to biological ones. For this reason, high attrition is a common problem in weight loss interventions with serious consequences for weight loss management and frustration (5). Understanding the factors contributing to attrition could allow the identification of patients at the highest risk of dropout, supporting them during the intervention, or identifying more suitable intervention options.

Previous studies have associated high attrition rates with many variables, such as demographics (age, age at the onset of obesity, sex, occupational status, education) (6–9), anthropometrics (body-mass index BMI) (9), psychological aspects (high weight loss expectations, health status, self-esteem, perception of one’s body image, social or family support, anxiety, depression) (7, 9–13), behavior (eating habits and behavior, binge eating, physical activity level, alcohol consumption, lack of motivation, stress, and smoking) (9), and treatment-related factors (early nutritional interventions, previous dietary treatments, type of treatments, initial response, and expectation of weight loss) (8, 13–16). A consistent set of predictors has not yet been identified because of the large variety of study settings and methodologies used.

The initial response to treatment has emerged among the predictive factors related to treatment (16, 17). In fact, in most cases, the dropout percentage increases if the initial weight loss is unsatisfactory for the patient. Patients discontinue the program in the first weeks (early dropout) because of “failure to achieve the expected goal” and “feeling frustrated and disappointed.”

Regarding the psychological profile of patients before treatment, the dropout rate is higher when there is a greater state of anxiety and depression or, in general, a compromised state of mental health. These factors correlate with a lack of trust in healthcare personnel, lower motivation to undertake the path, or greater difficulty tolerating possible failure (10).

According to a different position statement from the Obesity Management Task Force of the European Association for the Study of Obesity (18) and from the Brazilian Association for the Study of Obesity and metabolic syndrome (ABESO), Cognitive Behavioral Therapy (CBT) should be used for weight management in patients with overweight and obesity (class of recommendation I; level of evidence A) (18, 19). CBT is the oldest and most studied behavior change theory used in nutrition counseling. It provides a theoretical basis for most structured diet, exercise, and behavioral therapy programs. It is based on the premise of CBT theory that behavioral and emotional reactions are learned using cognitive and behavioral strategies. CBT focuses on external factors, such as environmental stimuli and reinforcement, and internal factors, such as thoughts and mood changes. CBT aims to help patients acquire specific cognitive and behavioral skills to increase adherence to the dietary and physical activity changes required for body weight management that can be used going forward to support their mental health and wellness.

Dietitians apply strategies on both factors to unlearn undesirable eating patterns and behaviors and replace them with more functional thoughts and actions (20, 21). CBT strategies include goal setting, self-monitoring, problem-solving, social support, stress management, stimulus control, cognitive restructuring, relapse prevention, rewards, and contingency management (20).

This study systematically reviewed the dropout rate and predictive factors of dropout in cognitive behavioral treatment (CBT) in adult patients with overweight or obesity in order to provide a comprehensive assessment of the literature about this topic.

## Materials and methods

This systematic review was performed based on Preferred Reporting Items for Systematic Reviews and Meta-Analyses (PRISMA) method (22). The following electronic databases were searched: PubMed, Scopus, and the Cochrane Library. The language used was English. Only full-text articles published in the last 20 years and full-text articles available were included.

Clinical and observational trials, case reports, and case series were included to investigate the dropout rate and predictive factors associated with the dropout rate in adults living with overweight and obesity undergoing cognitive behavioral therapy (CBT). *In-vitro* and animal studies, guidelines, letters, editorials, comments, news articles, conference abstracts, theses, and dissertations were excluded.

The study protocol was registered on the PROSPERO platform (registration number: CRD42022369995).

### Literature research strategy

An electronic search was conducted with subject index terms, including “patient dropouts,” “weight loss,” “diet reducing,” “weight loss therapy,” and “diet therapy.” Google Scholar was used to search for gray literature, and some references found in the review articles were included manually. The study population consisted of adults aged 18–65 years old with overweight or obesity (Body Mass Index (BMI) ≥25 kg/m2). The intervention was cognitive behavioral therapy (CBT), and the comparison was standard dietary treatment. The research question, and specific inclusion and exclusion criteria based on PICOS strategy are presented in Table 1.

**Table 1:** PICOS criteria of inclusion and exclusion.

PICOS criteria	Inclusion criteria	Exclusion criteria
Population	adult (18–65 years old) patients with overweight or obesity (BMI ≥ 25 kg/m^2^),	Underweight or normal weight, younger or older patients
Intervention	Cognitive Behavioral Treatment (CBT)	Medical weight loss intervention, such as bariatric surgery or pharmaceutical treatment
Comparison	Standard dietary treatment	Not applicable
Outcomes	Dropout rate and weight loss	Not related with dropout rate or weight loss
Types of Studies included	Observational trials and studies, case reports and case series	Not English language; Full text not available; without the outcomes of interest; reviews, opinion articles, guidelines, letters, editorials, comments, news, conference abstracts, theses, and dissertations; and *in vitro* or animal studies
Research question	Which are the factors associated with dropout in cognitive behavioral treatment (CBT) in adults living with overweight and obesity?	

From Liberati et al. (22).

### Study selection

Two authors (LCLN and FM) independently performed the research and study selection. The articles found in the electronic database were organized using the Mendeley reference manager and Rayyan software (23), following two steps: (1) reading the titles and abstracts, and (2) evaluation of the complete articles selected in the previous stage and inclusion of other studies present in the references of the selected articles. The decision to include the articles was based on the PICOS strategy: population (P) – adult (18–65 years) patients with overweight or obesity (BMI ≥ 25 kg/m^2^); intervention (I) – cognitive behavioral treatment; control (C) – standard dietary treatment; outcome (O) – attrition rate and weight loss; and study type (S) – clinical and observational trials, case reports, and case series. These inclusion criteria were used to identify potentially relevant abstracts, and if abstracts were coherent with them, full papers were obtained and assessed. In cases of disagreement, a third author (CF) reviewed the full-text articles to make a decision. Studies meeting the specified inclusion criteria were included in the qualitative analysis. Additionally, relevant articles were manually added to the search. Study sample characteristics, design, intervention, dropout rate, results, and quality were extracted. The risk of bias was assessed using the RoB 2.0 Cochrane tool (24), and the quality of evidence was assessed using the Mixed Methods Appraisal (MMAT) system (25).

## Results

**Figure 1:**
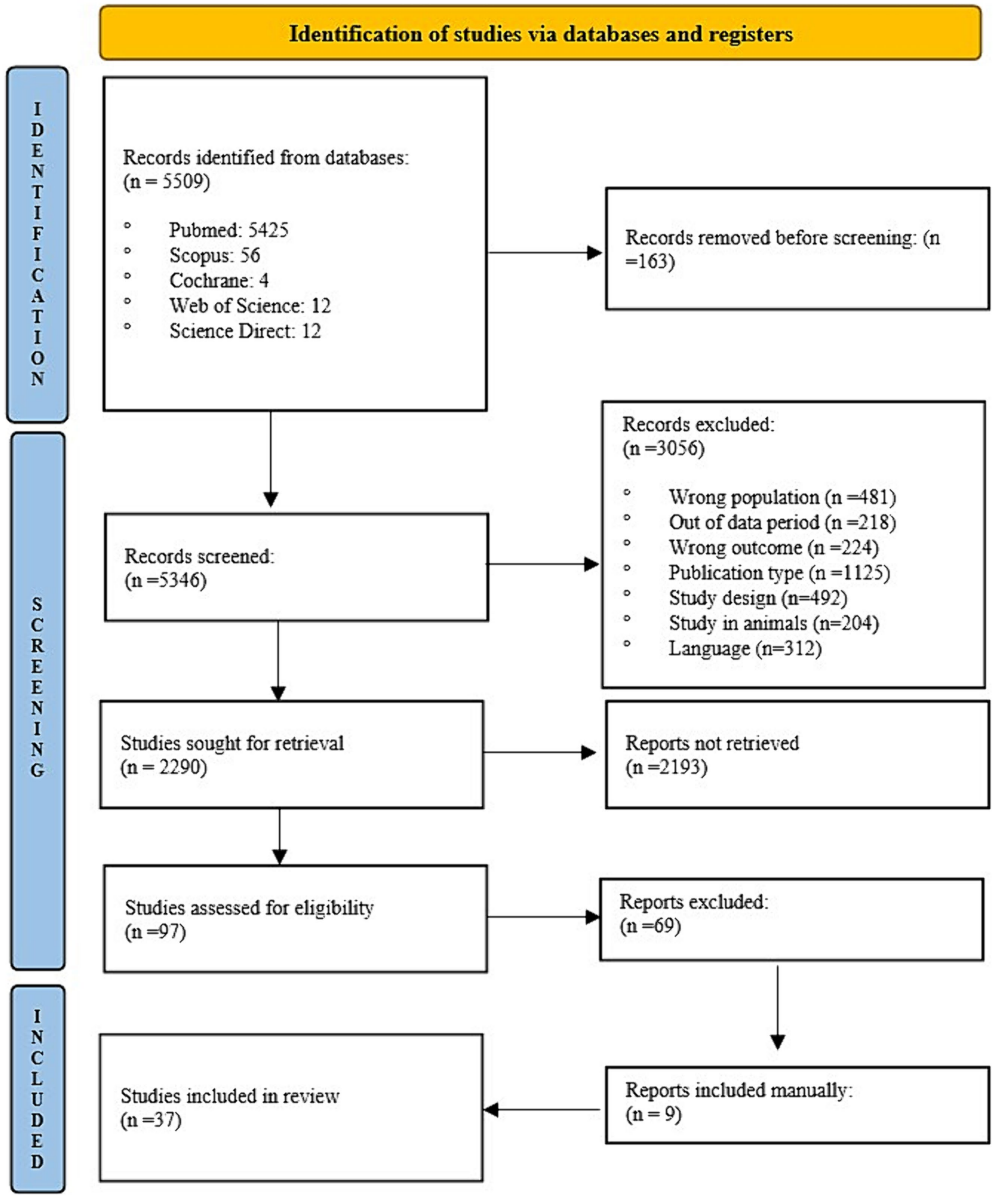
Flowchart of included studies (18). From: Page et al. (26).

After searching the databases using search strings, 5,509 articles were identified. Figure 1 describes the selection phase and the retained articles in each phase. After selection, 37 articles that used the CBT approach in weight loss interventions addressed the dropout rate and associated predictive factors. The details of each selected article are listed in Table 2.

**Table 2:** Description in details of the included articles, 2023.

Study, Year, Country	Type of study	Sample characteristics	Intervention	Dropout			Quality (MMAT)
				Predictive factors of dropout	Dropout rate and reasons	Results	
Teixeira et al., 2004, Portugal and USA (27)	Observational study	*n* = 158 F Age, y (Mean ± SD): 48.0 ± 4.5 BMI, kg/m^2^ (Mean ± SD): 31.0 ± 3.8	16 wk. lifestyle WL program (CBT) + randomization for online contact (yes or no) Follow-up: 1y Professionals: n.d.	DO: more previous WL attempts, poorer quality of life, more stringent weight outcome evaluations, lower reported carbohydrate intake at baseline. Completion: Predicted correctly in 84% of all cases.	DO rate: 30% (*n* = 47) DO reasons: Lack of time (35%), dissatisfaction with the program/staff (22%), personal life issues (17%), health limitations (17%).	Predictors of success (16 mo): more moderate weight outcome evaluations, lower level of previous dieting, higher exercise self-efficacy, and smaller waist-to-hip ratio. Success status: predicted correctly in 74%	***
Dalle Grave et al., 2005, Italy (28)	Observational multicenter study (QUOVADIS)	*n* = 1,000 785 F/215 M Age, y (Mean ± SD): 45.3 ± 11.1 (F); 45.0 ± 10.4 (M) BMI, kg/m^2^ (Mean ± SD): 37.5 ± 6.0 (F); 36.6 ± 5.5 (M)	Intensive CBT treatment period (3–6 mo), followed by less intensive continuous care (every 2–4 mo), Follow-up: 36 mo Professionals: n.d.	DO + disagreed with the treatment program: higher BMI. a higher maximum BMI, higher Expected One-Year BMI Loss DO + satisfactory results: lower BMI and a lower maximum BMI Completion: older, lower Expected One-Year BMI Loss.	DO rate: 20% (after the first visit), 58% (12 mo), 84.3% (36 mo) DO rate differs between centers, ranging from 61 to 98% DO reasons: logistics, unsatisfactory results and lack of motivation.	WL: 5.2% in completers vs. 3.0% in DO DO patients satisfied with the results or confident to lose additional weight without professional help reported a mean WL of 9.6 and 6.5%, respectively. Predictor for continuous care: lower Expected One-Year BMI Loss DO ↑ systematically for any 5% expected BMI loss.	****
Dalle Grave et al., 2005, Italy (29)	Observational study (QUOVADIS)	*n* = 1785 1,393 F/392 M Age, y (Mean ± SD): 44.8 ± 11.1 (F); 44.0 ± 10.7 (M) BMI, kg/m^2^ (Mean ± SD): 38.2 ± 6.3 (F); 38.0 ± 6.6 (M)	CBT, Follow-up: 12 mo Professionals: n.d.	DO (12 mo): age and expected 1-year BMI loss	DO rate at 1y: 51.7% (*n* = 923)	DO ↑ systematically for a unit increase in expected BMI loss at 1y Risk elevated in the first 6 mo.	****
Stahre et al., 2005, Sweden (30)	Randomized controlled trial	*n* = 105 F Age, y (Mean ± SD): 45.4 ± 9.8 (CBT); 45.2 ± 11.3 (Control) BMI, kg/m^2^ (Mean ± SD): n.d. BMI, kg/m^2^ ≥ 30	CBT 10 wk. (*n* = 62) vs. Control: wait-list group (*n* = 43) Follow-up: 18 mo Professionals: Social worker CBT specialized.	Not mentioned	Completion: 92% in the intervention group (*n* = 57) DO reasons (10 wk): practical reasons, not agree with the treatment method. DO rate at 18 mo: 40%	Mean WL: 8.5 kg (CBT) (SD = 16.1) vs. + 2.3 kg (SD = 7.0) (control). Significant weight difference between groups at the 18 mo follow-up	****
Bauer et al., 2006, Switzerland (31)	Randomized double-blind, placebo-controlled study	*n* = 73 68F/5M 29 with/44 without sBED Age, y (Mean ± SD): sBED 42.0 ± 13.8 (sibutramine); 40.1 ± 5.6 (placebo) no-BED 45.1 ± 10.1 (sibutramine); 40.5 ± 10.2 (placebo) BMI, kg/m^2^ (Mean ± SD): sBED 34.1 ± 3.8 (sibutramine); 35.9 ± 6.6 (placebo) no-BED 36.1 ± 4.8 (sibutramine); 36.3 ± 4.6 (placebo)	Sibutramine (10 mg/day for the first 4 wk. and 15 mg/day for the remaining 12 wk) or Placebo in CBT-WL program Follow-up: 16 wk. Professionals: Nutritionist and psychologist	Not mentioned	DO rate: 27.4%. DO reasons: personal reasons, medication side effects or absence in more than 4 group meetings. Random distribution of DO concerning sibutramine and sBED.	Higher WL in the sibutramine group, ↓ binge episodes in both groups.	****
Burke et al., 2006, USA (32)	Randomized controlled trial (PREFER)	*n* = 182 159 F/23 M Age, y (Mean ± SD): 44.1 ± 8.6 BMI, kg/m^2^ (Mean ± SD): n.d. BMI, kg/m^2^ (range): 27–43	SBT vs. SBT + LOV, 18 mo Follow-up: n.d. Professionals: n.d.	Not mentioned	Adherence to self-monitoring: 80% in yes + sCBT, 85% in yes + sCBT + LOV, 86% in no + sCBT, 84% in no + sCBT + LOV. Retention rate: 86.3% at 6 mo	Change scores (2 groups); carbohydrate and protein consumption, polyunsaturated-to-saturated fat ratio, LDL-C level. SBT-LOV (100% adherents): greater WL, total cholesterol, LDL-C, glucose and consumed less fat.	***
Grossi et al., 2006, Italy (33)	Observational study (QUOVADIS)	*n* = 940 727 F/213 M Age, y (Mean ± SD): 50.7 ± 10.7 (continuers); 48.7 ± 10.5 (DO) BMI, kg/m^2^ (Mean ± SD): 38.9 ± 7.0 (continuers); 38.5 ± 6.4 (DO)	Telephone interview to classify reasons for dropouts Follow-up: 41 mo Professionals: Psychologists, clinicians, epidemiologists	Completion: higher university education	DO rate (1y): 62%. DO rate (41 mo): 81.5% (*n* = 766). DO reasons: practical difficulties (45%), unsatisfactory results (14%), scarce motivation (12%), confidence in the ability to additional WL without professional help (9%), and other reasons.	WL, FM, % of subjects achieving a body weight loss>10% and/or a reduction in the FM >5% resulted significantly higher in NPPR group than in the diet-therapy group.	***
Mefferd et al.,2007, USA (34)	Randomized controlled trial	*n* = 85 F Breast cancer survivor Age, y (Mean ± SD): 56.3 ± 8.2 BMI, kg/m^2^ (Mean ± SD): 31.0 ± 4.2	Intervention group (CBT) vs. Wait-list group, 16 wk. of weekly sessions Professionals: n.d.	Not mentioned	DO rate: 10.6% (16 wk) DO reasons: clinical or mammographic evidence denoting breast cancer recurrence, family crisis, and loss of follow-up.	CBT: ↓ body weight, BMI, waist and hip circumference ↓total body fat (% and kg), trunk fat mass (kg), leg fat mass (kg) ↓ TG and total cholesterol/high-density lipoprotein cholesterol ratio.	***
Minniti et al., 2007, Italy (35)	Randomized clinical trial	*n* = 129 F Age, y (Mean ± SD): 49.5 ± 12.0 (completers); 45.1 ± 9.2 (non-completers) BMI, kg/m^2^ (Mean ± SD): 35.6 ± 4.0 (completers); 36.2 ± 6.4 (non-completers)	IT (*n* = 72) or GT (*n* = 57) Follow-up: 6 mo Professionals: Physicians, psychologist, dietitian	Completers: older, worse BUTa General Severity Index score than non-completers.	DO rate: 37,2%, higher in IT = 54,2% vs. GT = 15,8%. There was Δ in DO rate	Completers were older and had significantly higher scores in BUTa GSI (1.22 ± 0.64 vs. 1.02 ± 0.62). WL in completers: 6.39 ± 4.63% of initial weight	***
Stahre et al., 2007, Sweden (36)	Randomized controlled trial	*n* = 54 F child-care professionals Age, y (Mean): 48.5 BMI, kg/m^2^ (Mean): 36.6	10 wk.: CBT vs. Behavioral program with physical activity Follow-up: 10 wk. + 18 mo Professionals: Social worker specialized in conservatively treatment, occupational doctor and nurse	Not mentioned	DO rate: 44% in CBT vs. 26% (control) at the end of the treatment. 87% of completers in CBT vs. 80% in the control group at 18 mo follow-up. DO reasons: work, on sick leave and unknown reasons	WL (end of treatment): 8.6 kg (CBT) and 0.7 kg (control).	****
Befort et al., 2008, USA (37)	Pilot randomized controlled trial	*n* = 44 F Afro American Age, y (Mean ± SD): 44.3 ± 11.6 BMI, kg/m^2^ (Mean ± SD): 39.8 ± 6.4	Behavioral WL program +: - MI (Motivational Interviewing) vs. - HE (Health Education) Professionals: Doctorate-level psychologist and a Masters-level counselor or dietitian	Not mentioned	DO rate: 22.7% DO reasons: lost to follow-up.	No significant differences between MI and HE in adherence or treatment outcome: completion of individual sessions did not differ	***
Lowe et al., 2008, USA (38)	Randomized controlled trial	*n* = 103 F Age, y (Mean ± SD): 43.9 ± 10.5 BMI, kg/m^2^ (Mean ± SD): 31.9 ± 2.6 61.2% whites, 35.9% African Americans, 2.9% Asians	WL phase (8 wk), WL maintenance (14 wk): CBT (CG) vs. CBT + EFMA vs. CBT + EFMA + REDE Professionals: n.d.	DO: significantly more likely to be African Americans than whites and more likely not to have a college degree	DO rate: 12% (9 wk), 22% (post-intervention), 31% (6 mo), 40% (18 mo).	WL: 7.6 ± 2.6 kg during WL phase and 1.8 ± 2.3 kg during WM phase. No Δ among groups and all groups regained weight between 6–18 mo follow-up.	****
Brambilla et al., 2009, Italy (39)	Randomized controlled trial	*n* = 30 F with BED Age, y (Mean ± SD): Group 1: 47.0 ± 8.0 Group 2: 45.0 ± 11.0 Group 3: 46.0 ± 8.0 BMI, kg/m^2^ (Mean ± SD): Group 1: 39.0 ± 6.0 Group 2: 34.0 ± 6.0 Group 3: 34.0 ± 5.0	Group 1 (1700 kcal diet + CBT + sertraline and topiramate) vs. Group 2 (1700 kcal diet + CBT + sertraline) vs. Group 3 (nutritional counseling + CBT) Follow-up: 6 mo Professionals: Nutritionists, psychiatrists	Not mentioned	DO rate: 16% DO reasons: lack of motivation, family problems	↓ BMI, weight. Binge episode frequency in group 1.	**
Dalle Grave et al., 2009, Italy (40)	Longitudinal observational study (QUOVADIS)	*n* = 500 394 F/106 M Age, y (Mean ± SD): 46.2 ± 10.8 BMI, kg/m^2^ (Mean ± SD): 37.3 ± 5.6	12 mo WL treatment (CBT) Professional: Physicians	Not mentioned	DO rates were significantly different among centers. During the study, a large DO rate was observed.	Successful WL was associated with ↑ dietary restraint and ↓ disinhibition.	****
Donini et al., 2009, Italy (41)	Prospective trial (non-randomized)	*n* = 464 380 F/84 M Age, y (Mean ± SD): 46.4 ± 12.0 (NPPR); 45.1 ± 13.0 (N) BMI, kg/m^2^ (Mean ± SD): n.d.	Standard diet vs. NPPR (physical activity + CBT). Follow-up: duration of the treatment was not fixed in advance. Professional: Dietician and psychotherapist	Not mentioned	DO rate: 5.5% in NPPR vs. 54.4%	↓ weight and BMI higher in NPPR NPPR treatment duration was higher	***
Forlani et al., 2009, Italy (42)	Prospective cohort observational survey	*n* = 822, T2DM patients 413 F/409 M Age, y (Mean ± SD): 64.8 ± 10.3 (Diet) 62.4 ± 9.8 (ENE) 56.7 ± 8.5 (CBT) BMI, kg/m^2^ (Mean ± SD): n.d.	Diet vs. ENE (4 sessions) vs. CBT (12 and 15 sessions) Follow-up: 4y observation, Professionals: Dietitian, physician, psychologist	Not mentioned	DO rate: less than 5% (2y), 7%, (4y), not different among groups.	Higher WL in CBT. ENE and CBT associated with ↓ risk of *de novo* insulin treatment	***
Werrij et al., 2009, Netherlands (43)	Randomized controlled trial	*n* = 200 162 F/38 M Age, y (Mean ± SD): 45.0 ± 12.0 BMI, kg/m^2^ (Mean ± SD): 33.4 ± 4.6	CDT (diet + CBT) vs. EDT (diet + exercise), 10wk sessions Follow-up: 1y Professionals: Dietitian, cognitive behavior therapist, physiotherapist	DO had higher pretreatment scores on weight concerns, shape concerns, eating psychopathology, and depression.	DO rate: 21% Higher DO in EDT (26%) than in the CDT (16%), predicted by higher pretreatment eating psychopathology (EDE-Q global scores) and by specific treatment	WL: ↓1.36 BMI points CDT vs. ↓ 1.44 BMI points EDT in short-term; ↓ 1.35 BMI points CDT vs. ↓ 1.08 BMI points EDT. EDT group regained 25% of weight lost, CDT group no.	****
Garaulet et al., 2010, Spain (44)	Explanatory study	*n* = 454 380 F/74 M Age, y (Mean ± SD): 39.2 ± 11.2 (completers); 39.3 ± 12.1 (non-completers) BMI, kg/m^2^ (Mean ± SD): 30.2 ± 4.8 (completers); 32.7 ± 5.4 (non-completers)	Behavioral weight-reduction program, Follow-up: 12 mo Professionals: n. d.	DO: significantly more obese, significantly greater barriers-to-weight-loss score, more frequent stress with dieting and planned eating less frequently than those who successfully finished the treatment	DO rate: 47.3%	-	***
Makoundou et al., 2010, Switzerland (45)	Explanatory study	*n* = 50 Age, y (Mean ± SD): n.d. BMI, kg/m^2^ (Mean ± SD): 35.7 ± 0.9	Maintenance multifactorial approach (diet + CBT + orlistat ‘on–off’) Follow-up: 2y Professionals: Physician, dietitian	Not mentioned	DO rate: 12% DO reasons: did not cooperate, failed to return at 2y, underwent a surgical operation for a ring implant.	29 completers (65%) with no relapse vs. 15 with relapse (35%). At 2y body weight remained stable and among all subjects 58% experienced additional WL, while 42% had at least one episode of weight regain during 2y follow-up.	***
Buscemi et al., 2011, Italy – Lebanon and USA (46)	Cohort study	*n* = 251 Age, y (Mean ± SD): 41.2 ± 3.7 (success group); 40.5 ± 1.6 (failure group) BMI, kg/m^2^ (Mean ± SD): 35.9 ± 2.6 (success group); 33.2 ± 0.8 (failure group)	Medical Nutritional Treatment (MNT) with CBT Follow-up: 10y Professional: Dietitian	Not mentioned	DO rate: 39%	Completers: 73.2% successful WL (6 mo). 1 year: Success: WL 9.8% (−9 ± 0.4 kg) Failure: WL 3.1% (−2.7 ± 0.2 kg)	***
Christensen et al., 2012, Denmark (47)	Cluster randomized single-blinded controlled trial (FINALE-health)	*n* = 98 F health care workers Age, y (Mean ± SD): n.d. BMI, kg/m^2^ (Mean ± SD): 30.7 ± 5.4 (intervention group); 30.4 ± 4.9 (control group)	Intervention group (diet, physical activity and CBT training) or control group, during working hours 1 h/wk. Follow-up: 12 mo Professional: n.d.	Not mentioned	DO rate: 15% DO reasons: left company, long term sick, withdrew	WL (intervention group): −6 kg, BMI (intervention group): −2.2 points, Fat mass (intervention group): −2.8%	*****
Göhner et al., 2012, Germany (48)	Quasi-experimental design	*n* = 316 245 F/71 M Age, y (Mean ± SD): 50.6 ± 10.8 BMI, kg/m^2^ (Mean ± SD): 34.7 ± 3.1	IG with M.O.B.I.L.I.S program (190) vs. CG (126), Follow-up: 2y Professional: n.d.	Not mentioned	DO rate: 4.8% (intervention group, first 6 mo) DO reasons: illness or injury, dissatisfaction with the program, excessive strain, vocational, or private changes, unknown reasons.	Significant decrease in BMI and WL in the intervention group. Increased physical activity level in the intervention group. No significant differences between the groups 2y after baseline.	****
Buscemi et al., 2013, Italy (49)	Cohort study	*n* = 251 Age, y (Mean ± SD): n.d. BMI, kg/m^2^ (Mean ± SD): n.d.	Medical Nutritional Treatment (MNT) with CBT Follow-up: 10 y Professional: Dietitian	Not mentioned	DO rate: 64.9%	No significant predictors of the 10y BW change including as covariates age, gender smoking, initial BMI, HADS and DRT items scores	***
Dalle Grave et al., 2013, Italy (50)	Randomized controlled trial	*n* = 88 51 F/37 M Age, y (Mean ± SD): 47.6 ± 11.1 BMI, kg/m^2^ (Mean ± SD): 45.6 ± 6.7	High Protein Diet (HPD) + CBT vs. High Carbohydrate Diet (HCD) + CBT Follow-up: 3 wk. inpatient and 48 wk. outpatient Professionals: Dietitians, physician and psychologist	Not mentioned	DO rate in both groups: 25.6%. DO rate (wk 15): 21.6% no differences in DO rates between groups HPD had higher DO rates at wk. 27, but lower rates between wk. 27 and study end compared to HCD group	WL in HPD: 15%, WL in HCD: 13.3% at 1y. Both diets produced a similar improvement in secondary outcomes	*****
Michelini et al., 2014, Italy (51)	Randomized controlled trial	*n* = 146 109 F/37 M Age, y (Mean ± SD): 45.0 ± 10.8 BMI, kg/m^2^ (Mean ± SD): 32.3 ± 3.7	Standard Care group (*n* = 73) or CBT + diet group (*n* = 73) Follow-up: 6 mo Professionals: Physician, psychologist, dietitian	DO reasons: high level of stress (GHQ-28 score within VCAO test)	DO rate: 30.1% (39.7% in CBT and 24.7% in Standard Care group), with no significant difference DO reasons: objective reasons (pregnancy, acute illness and unforeseen job difficulties)	High level of stress appears to be the most important predictor of dropout	***
Tagliabue et al., 2015, Italy (52)	Nested case–control study	*n* = 59 F (20 cases vs. 39 controls) Age, y (Mean ± SD): 42.2 ± 10.4 (cases); 42.4 ± 14.0 (controls) BMI, kg/m^2^ (Mean ± SD): 36.1 ± 4.4 (cases); 35.6 ± 5.1 (controls)	CBT (50 min individual sessions) vs. standard diet Follow-up: 6 mo Professional: Psychologist and registered dieticians, 6 months	DO reasons: lack of motivation, personal family reasons, lack of achievement of satisfactory WL DO: age at first diet attempt (treatment) and SCL-90 anger-hostility sub scale (controls)	DO rate: 35% in cases, 62% in controls.	CBT was significantly more effective in dropout reduction, without no differences in WL	*****
Dalle Grave et al., 2015, Italy (53)	Observational study (QUOVADIS II)	*n* = 634 F Age, y (Mean ± SD): BMI, kg/m^2^ (Mean ± SD):	Programs of 8 centers: including dieting, CBT and drugs Follow-up: 12 mo Professional: Medical doctor	DO: higher baseline weight and with younger age; higher percent weight targets, with the notable exception of dream and happy weight	DO rate: 32.3% at 1y	DO was associated with more challenging, acceptable and disappointing weight targets, but not with dream and happy weight goals.	****
Calugi et al., 2016, Italy (54)	Prospective case–control study	*n* = 108 F (54 with BED, 54 without BED) Age, y (Mean ± SD): 40.2 ± 13.6 BMI, kg/m^2^ (Mean ± SD): 39.7 ± 6.1	Residential CBT program, 6 mo Follow-up: 5y Professional: experts in the field and clinical psychologists	Not mentioned	DO rate: 19.5% (6 mo).	Similar WL (at 6 mo and at 5y) and improved psychological variables in both groups, but higher impairment in BED at 6 mo. At 5y follow-up more than half of the BEDs were no longer classifiable as having BED.	****
Sawamoto et al., 2016, Japan (55)	Part of a randomized controlled trial	*n* = 119 F Age, y (Mean ± SD): 47.7 ± 1.2 (completers); 43.9 ± 2.1 (non-completers) BMI, kg/m^2^ (Mean ± SD): 31.3 ± 0.5 (completers); 32.0 ± 0.9 (non-completers)	CBT for WL: (1) 7 mo, (2) 3 mo (maintenance) Follow-up: 2 y (if previous loss >5% initial weight) Professionals: Doctors and certified nutritionist	DO: stronger body shape concern, tended to not have jobs, perceived their mothers to be less caring, and were more disorganized in temperament	DO rate: 24,4% Most dropped out in the first 3 mo (62.0% of the total dropouts).	Best DO predictor: shape concern.	***
Calugi et al., 2017, Italy (56)	Randomized controlled trial	*n* = 88 51 F/36 M Age, y (Mean ± SD):46.7 ± 11.1 BMI, kg/m^2^ (Mean ± SD): 45.6 ± 6.7	High Protein Diet (HPD) + CBT vs. High Carbohydrate Diet (HCD) + CBT, 51 wk. (27 wk. WL phase, 24 wk. WM) Professional: physicians, registered dietitians, and psychologist	DO: % WL necessary to reach dream and happy WL goals	DO rate (WL phase): 11.4%	Similar WL expectation and satisfaction between two groups. Expected WL (kg), but no WL (%) predicted WL. Both satisfaction and WL (kg) in kg were independent predictors of WM.	****
Figura et al., 2017, Germany (57)	Observational pre-post study	*n* = 102 75 F/27 M Age, y (Mean ± SD): 45.8 ± 10.8 (LSG); 50.6 ± 11.3 (control) BMI, kg/m^2^ (Mean ± SD): 51.4 ± 8.1 (LSG); 40.3 ± 6.7 (control)	LSG group (Laparoscopic Sleeve Gastrectomy) (*n* = 62) vs. Control group (diet, exercise and CBT) 1y (*n* = 40) Follow-up: 19 mo Professionals: Psychologist or physician specialized in psychosomatic medicine, surgeon, endocrinologist, nurse, dietitian and psychotherapist.	Not mentioned	DO rate: 30% in Laparoscopic Sleeve Gastrectomy, 34% in CT.	WL in Laparoscopic Sleeve Gastrectomy: 25.9 kg, WL in CT: 5.4 kg. BMI in Laparoscopic Sleeve Gastrectomy: −7.8 kg/m^2^, BMI in CT: −7.2 kg/m^2^.	****
Sasdelli et al., 2018, Italy (58)	Observational study	*n* = 793 543 F/250 M Age, y (Mean ± SD): 48.7 ± 13.5 BMI, kg/m^2^ (Mean ± SD): 40.8 ± 7.7	Group-based CBT and psychological questionnaires, 3 mo Follow-up: 24 mo Professional: n.d.	DO was significantly favored by the presence of anxiety and depression in F, not in M, and was significantly reduced by concern for present health (at 6-month, with a non-significant effect in the long term), whereas it was favored by body image dissatisfaction or by considering CBT as a temporary step to bariatric surgery. Short-term DO was driven by more challenging targets, not by dream weight targets.	DO rate: 12% (3 mo), 24% (6 mo), 41% (12 mo), 55% (24 mo). At 6 mo DO was higher in F (27 vs. 17%); but no gender Δ at 12 mo (43 vs. 36%) and 24 mo (55 vs. 54%)	WL: 5.8 kg ± 7.1 kg (−4.8%) at 6 mo. WM > 10% at 24 mo (32% of C): 17%	****
Galindo Munoz et al., 2019, Spain (59)	Randomized clinical trial	*n* = 120 90 F/30 M Age, y (Mean ± SD): n.d. BMI, kg/m^2^ (Mean ± SD): n.d.	Cognitive Training Intervention (hypocaloric diet +12 cognitive training sessions via Brain Exercise) or CBT as control group (hypocaloric diet +30 min sessions) Follow-up: 12 wk. Professional: Dietitians	Not mentioned	DO rate: 20% No Δ between groups in DO rate. DO reasons: lack of adherence to the intervention.	Total WL (%) and Δ anthropometric were higher in Cognitive Training Intervention, while biochemical parameters improved in both groups. All cognitive measures improved in Cognitive Training Intervention.	*****
Dalle Grave et al., 2020, Italy (60)	Observational study	*n* = 67 51 F/16 M Age, y (Mean ± SD): n.d. BMI, kg/m^2^ (Mean ± SD): 39.8 ± 5.8	CBT-OB 22 sessions (14 in 6 mo WL phase, 8 in 12 mo WM phase), Professionals: Physician specialized in clinical nutrition and in nursing	Not mentioned	DO rate (WL phase): 13.4% DO rate (WM phase): 10.44%	WL: 11.5% (10% in the intention to treat analysis) at 6 mo and 9.9% (7.5% in the intention-to-treat analysis) at 18 mo. WL: ↓ cardiovascular risk factors, anxiety, depression and eating disorder psychopathology, and with an improvement in obesity-related quality of life.	*****
Calugi et al., 2021, Italy (61)	Retrospective case–control study	*n* = 258 180 F/78 M Age, y (Mean ± SD): 57.0 ± 14.2 (lockdown group); 56.5 ± 14.0 (control) BMI, kg/m^2^ (Mean ± SD): 41.6 ± 8.3(lockdown group); 42.2 ± 8.1 (control)	CBT-OB (Low Calorie Diet + physical activity + group sessions) + telephone interview, 21d + 6 mo follow-up Professional: n.d.	Control: respondents > age to follow-up interview (respondents) 59.6 (SD = 10.8) years *VS* non-respondents 51.9 (SD = 16.9) years	DO rate: 45% (intervention) DO rate: 40% (control). DO reasons: refused telephone contact; not found or furnished unreliable data	WL > 9% and ↓ BED episodes in both groups. Lower WL in lockdown patients.	****
Gasparri et al., 2022, Italy and Bahrain (62)	Prospective cohort study	*n* = 168 117 F/61 M Age, y (Mean ± SD): 58.5 ± 13.0 BMI, kg/m^2^ (Mean ± SD): 41.3 ± 6.0	Multidisciplinary Residential Program (MRP) on WL, 8 w, 1y follow-up (2, 6, 24 mo after discharge). Professionals: Expert dietitian in CBT, physiotherapist (physical activity)	Not mentioned	Achieving a good WL goal during the rehabilitation program involves maintaining a lower weight afterwards without increasing the risk of DO	Total Mass: −5,68 kg, Fat Mass: – 4.42 kg, Fat Mass Index: −1724.56, Visceral Adipose Tissue: −0.3 kg, Arm Circumference: −1.63 cm, Calf Circumference: −1.16 cm, Free Fat Mass: – 1.24 kg. Improvement in glycaemic and lipid profile	****
Jiskoot et al., 2022, Netherlands (63)	Controlled clinical trial	*n* = 183 F with Polycystic Ovary Syndrome (PCOS) Age, y (Mean ± SD): 29.1 ± 4.4 BMI, kg/m^2^ (Mean ± SD): 34.0 ± 4.4 (LC); 34.7 ± 4.9 (LC + SMS); 32.7 ± 5.1 (UC)	Lifestyle Counseling (LC, 20 sessions involving CBT) vs. Lifestyle Counseling + SMS (LC + SMS, 20 sessions involving CBT and SMS) vs. Usual Care (UC). Follow-up: 12 mo Professionals: Dietician, psychologist, physical therapist	DO: higher baseline weight, participation in LC with SMS, and higher levels of androstenedione Completion: Participation in the CG and smoking was associated with lower odds of DO.	No Δ in DO rates between groups: 60.0% (control), 73.4% (LC), and 57.2% (LC + SMS). Overall DO rate: 63.4%	Depression and eating behavior were associated with ≥5% of WL.	****

F, female; M, male; y, years old (range or average); mo, months; wk, week; d, days; Δ, difference; BMI, Body Mass Index; CBT, Cognitive Behavioral Therapy; DO, Drop Out; WL, Weight Loss; WM, Weight Maintenance; FM, fat mass; SBT, Standard behavior treatment; LOV, Lacto-ovo Vegetarian; IT, Individual Nutritional Counseling; GT, (Cognitive Behavioral) Group Therapy; EFMA, Enhanced Food Monitoring Accuracy; REDE, Reduced Energy Density Eating; TFEQ, Three Factor Eating Questionnaire; ENE, Elementary Nutritional Education; sBED, subclinical Binge Eating Disorder; NPPR, Nutrition Psycho-Physical Reconditioning; GRWQ, Goals and Relative Weights Questionnaire; BUT, Body Uneasiness Test; SCL-90, Symptom CheckList; BES, Binge Eating Scale; HADS, Hospital Anxiety and Depression Scale; DRT, Dieting Readiness Test; PDQ-4-R, Personality Diagnostic Questionnaire-4-Revised; LDL-C, Low-density Lipoprotein-Cholesterol; SCL-90, Symptom Checklist-90; TG, triglycerides.

As shown in Table 2, the articles were categorized based on their origin, as follows: 20 studies were from Italy (54%), 6 from the USA, 2 from Germany, 2 from Sweden, 2 from Switzerland, 2 from Spain, 2 from the Netherlands, and the other countries with one article each (Denmark, Japan, Portugal, Lebanon, Bahrain). The selected sample, according to the inclusion criteria, comprised adult patients (aged 18–65 years) with overweight or obesity (BMI ≥25 kg/m^2^). During the analysis, it emerged that the predominant gender was female. The samples included in the various studies did not exhibit any other associated pathologies, except in some specific studies. For example, some studies have included breast cancer survivors (34), patients with polycystic ovary syndrome (PCOS) (25), patients diagnosed with T2DM (34), and patients with binge eating disorder (BED) (31, 39, 54).

Overall, dropout rates were between 5 to 62%. Dropout results were associated with several predictive factors, including demographic and psychological variables. In terms of demographic variables, some studies showed that younger age (35, 61), educational status (38), unemployed status (55), and ethnicity (38) could influence the dropout rate. Regarding psychological factors, attrition has been correlated with a higher expectation of 1-year BMI loss (28), weight and shape concerns (43, 55), body image dissatisfaction (58), higher stress (51), anxiety (58) and depression (43, 58). Other studies have associated an increased dropout rate with an increasing number of previous weight loss attempts (27), age at first diet attempt (52) and perceived stress with dieting (44). In the study by Sasdelli et al. (58), the dropout rate decreased with an increase in concern for present health, motivation, and consciousness about the importance of physical activity. The most frequent predictive factors are age and baseline BMI/weight referred to in 31 and 19% studies, respectively. Table 3 presents all the reported predictive factors analyzed in the selected studies.

**Table 3:** Reported predictive factors to dropout in the selected articles with CBT in patients with overweight or obesity.

Predictive factors	Teixeira et al., 2004 (27)	Dalle Grave et al., 2005 (28)	Dalle Grave et al., 2005 (29)	Grossi et al., 2006 (33)	Minniti et al., 2007 (35)	Lowe et al., 2008 (38)	Werrij et al., 2009 (43)	Garaulet et al., 2010 (44)	Michelini et al., 2014 (51)	Dalle Grave et al., 2015 (53)	Tagliabue et al., 2015 (52)	Sawamoto et al., 2016 (55)	Calugi et al., 2017 (56)	Sasdelli et al., 2018 (58)	Calugi et al., 2021 (61)	Jiskoot et al., 2022 (63)	TOTAL
Age		X	X		X					X					X		5
Age at first diet attempt											X						1
Education				X		X											2
Employed status												X					1
Life quality	X																1
Expected 1-year BMI loss		X	X														2
Weight concern							X										1
Shape concern							X					X					2
Body image					X									X			2
Stress									X								1
Anxiety														X			1
Depression							X							X			2
SCL-90 anger-hostility subscale											X						1
Eating psychopathology							X										1
Baseline BMI/weight								X		X						X	3
WL (%) to dream or happy weight													X				1
Previous attempts	X																1
Stress with dieting							X										1
Organization								X									1
Concern for health														X			1
Androstenedione levels																X	1
TOTAL	2	2	2	1	2	1	5	2	1	2	2	2	1	4	1	2	

CBT, cognitive behavioral treatment; BMI, body mass index; WL, weight loss; SCL-90, Symptom Checklist-90.

Common reasons for dropping out are reported in Table 4. Generally, the articles reported objective reasons (27, 36, 48, 51), such as long-term sickness, acute illness, pregnancy, logistics (28, 30), poor job conditions or job difficulties (36, 51, 55), low level of organization (55), dissatisfaction with initial results (27, 28, 31, 48, 52), lack of motivation (28, 39, 48, 52) and lack of adherence (59).

**Table 4:** Motivations to dropout in CBT patients with overweight or obesity, according to selected articles reporting this aspect.

Motivations	Lack of time	Lack of motivation	Lack of adherence	Dissatisfaction with the result or treatment	Personal issues	Health	Logistic or practical difficulties	Total
Teixeira et al., 2004 (27)	X			X	X	X		4
Stahre et al., 2005 (30)							X	1
Dalle Grave et al., 2005 (29)				X			X	2
Bauer et al., 2006 (31)	X			X				2
Grossi et al., 2006 (33)		X		X			X	3
Mefferd et al.,2007 (34)						X		1
Stahre et al., 2007 (36)					X	X		2
Brambilla et al., 2009 (39)		X			X			2
Göhner et al., 2012 (48)				X	X	X		3
Christensen et al., 2012 (47)					X	X		2
Michelini et al., 2014 (51)					X	X		2
Tagliabue et al., 2015 (52)		X		X	X			3
Galindo Munoz et al., 2019 (59)			X					1
Total	2	3	1	6	7	6	3	

CBT, cognitive behavioral treatment; BMI, body mass index; WL, weight loss.

Twenty-three of the included studies used CBT as the only approach (27–29, 32, 33, 35, 37–40, 44–46, 49–51, 53–56, 58, 60–62), three as a control group (31, 57, 59), and eleven as an intervention group (30, 34, 36, 41–43, 47, 48, 51, 52, 63, 64). Different strategies were used for the intervention group (IG) in trials where CBT was the control group. In the study by Muñoz et al. (59) IG was characterized by a Cognitive Training Intervention, which consisted of a hypocaloric diet and 12 cognitive training sessions via Brain Exercise (59), while in other studies by Laparoscopic Sleeve Gastrectomy (LSG) (57) or sibutramine administration (31). Most trials were conducted by dietitians or certified nutritionists, often CBT experts. Other professionals often involved included physicians, psychologists, and physical therapists.

In addition to analyzing dropout rate and weight loss, biochemical parameters (32, 34, 42, 59, 60, 62), such as glycaemic and lipid profiles, or cognitive and psychological variables, were assessed using specific questionnaires, such as the Goals and Relative Weights Questionnaire (GRWQ), Body Uneasiness Test (BUT), Symptom Checklist (SCL-90) and Binge Eating Scale (BES) (31, 35, 39, 40, 48, 49, 52, 54, 57, 59, 60, 62, 63).

**Figure 2:**
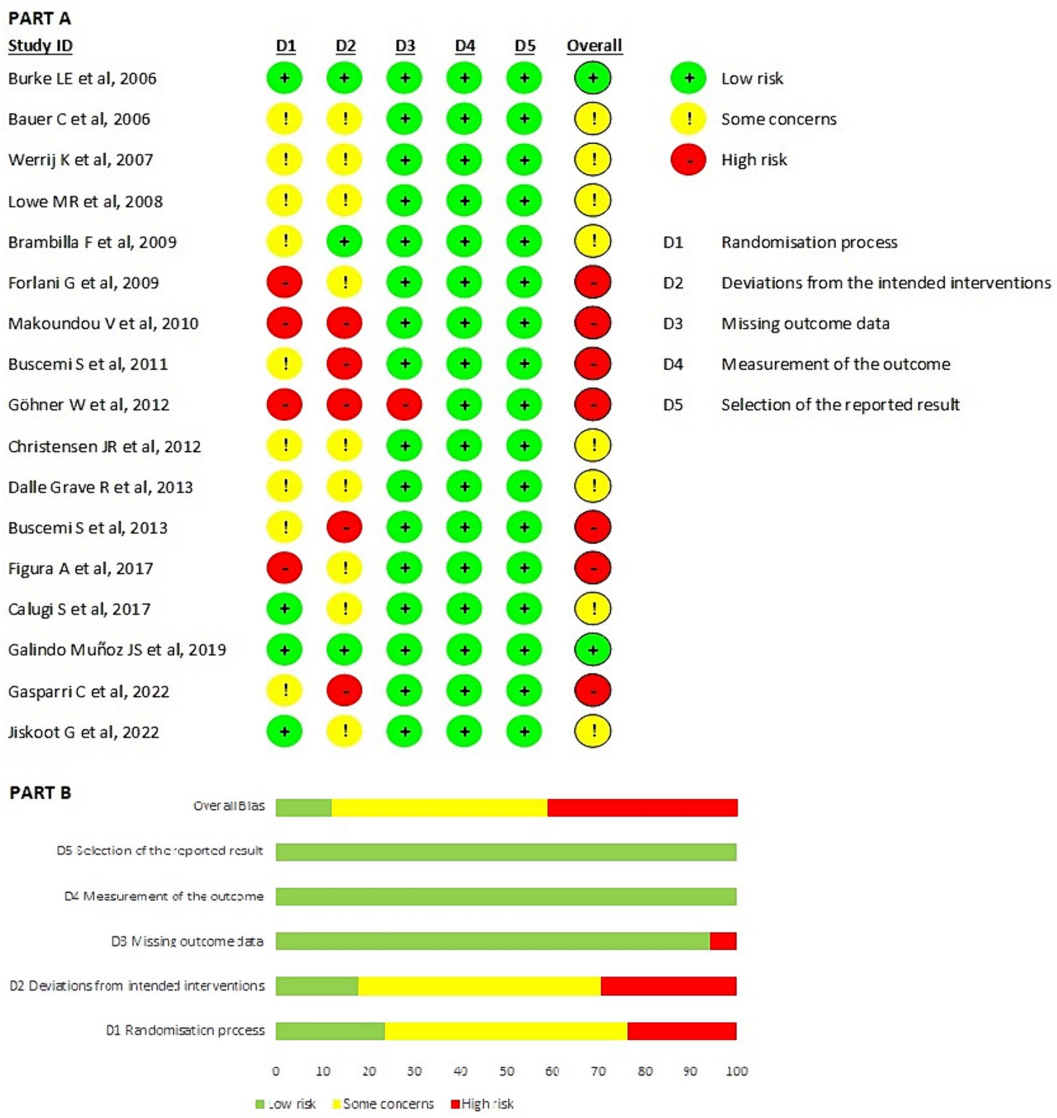
Results of risk of bias analysis of intention-to-treat studies (24). **(A)** Risk of Bias by article included on each domain. **(B)** Overall risk of Bias percentage on each domain. From Sterne et al. (65).

**Figure 3:**
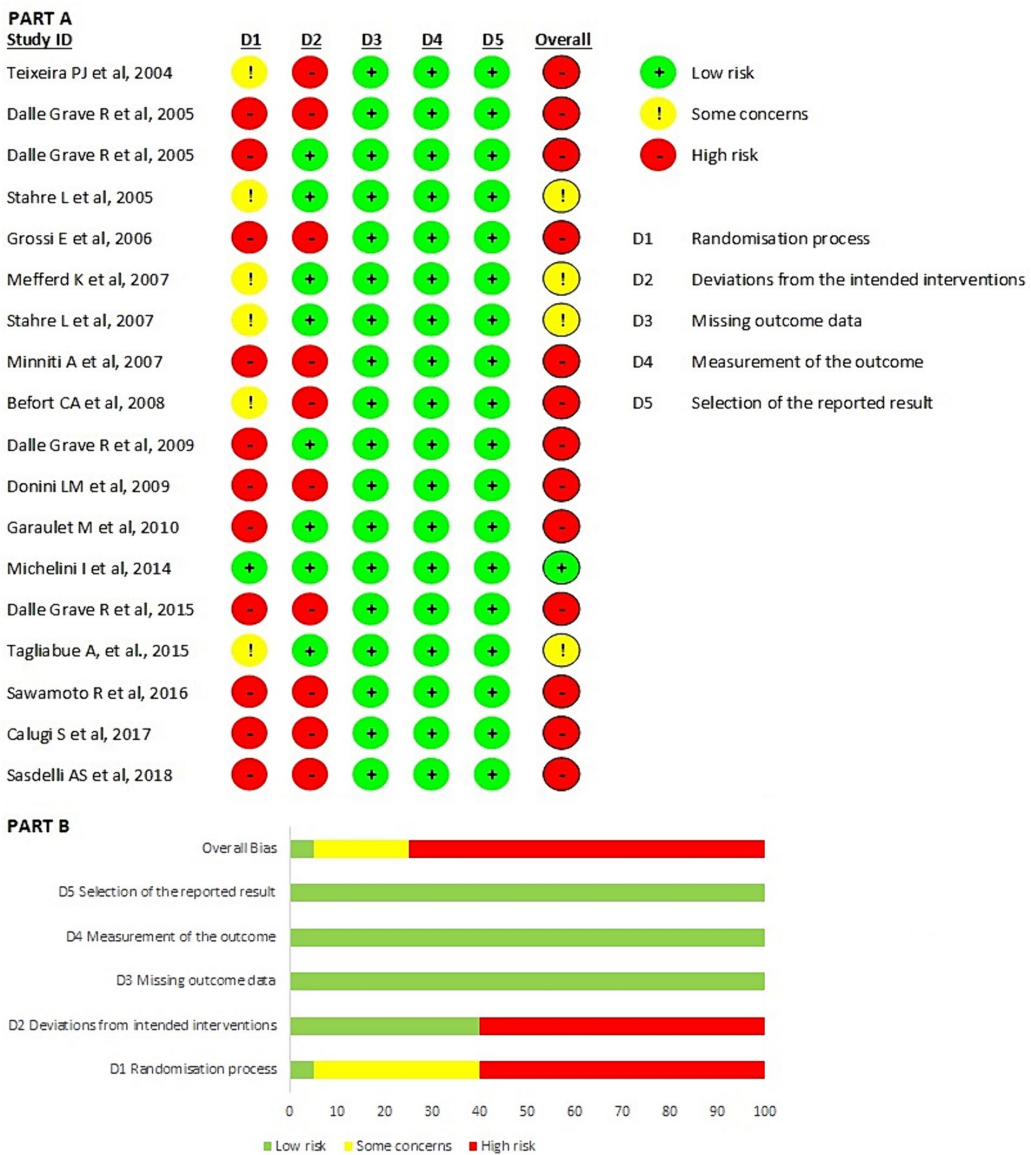
Results of risk of bias analysis of per protocol studies (24). **(A)** Risk of Bias by article included on each domain. **(B)** Overall risk of Bias percentage on each domain. From Sterne et al. (65).

According to the MMAT quality analysis, 13.5% of articles were classified as 5 stars, and none received the lowest quality grade (1 star). The majority of articles were classified as 4 stars (46%). Seventeen studies were included in the intention-to-treat analysis (Figure 2), and 20 studies were included in the per-protocol analysis (Figure 3). The highest domains with risk of bias in both the intention-to-treat and per-protocol analysis were domain 1, which pertained to the randomization process, and domain 2, which addressed deviations from the intended interventions. All other domains (D5: selection of the reported result, D4: measurement of the outcome; and D3: missing outcome data) had a low risk of bias. The overall valuation in the intention-to-treat analysis revealed 7 articles at high risk of bias (40%), while in the per-protocol analysis, there were 15 articles (75%). Also, in the intention-to-treat analysis, two articles (11.8%) were at low risk of bias, while in the per-protocol, there was one. In summary, more than half of the selected articles had a high risk of bias. The domain characterized by a higher level of bias was randomization, which was caused by the type of study design.

## Discussion

This study systematically reviewed the dropout rate and predictive factors associated with dropout from cognitive behavioral treatment (CBT) in adult patients with overweight or obesity. The review demonstrated that the dropout rate ranges between 5 and 62%. As indicated in the results, some demographic and psychological factors could be the predictors.

Most included studies considered psychological variables, revealing a significant association with the dropout rate. These findings support the hypothesis that analyzing the psychological profile of patients with overweight or obesity through specific questionnaires can help identify individuals at higher dropout risk. Furthermore, such assessments can assist in providing appropriate support during the intervention or determining suitable intervention.

There were common reasons among these studies for patients to discontinue treatment. Some of these issues, such as organization and logistics, can be resolved by providing practical tools to address the barriers to successful treatment. Others, such as dissatisfaction with initial results and lack of motivation, could be managed by helping patients to understand that an improvement in general health is already obtained with a weight loss of 5–10% of the initial weight, thereby reducing their weight loss expectations and weight loss targets. According to Dalle Grave et al., regardless of the degree of weight loss, people with obesity have a high prevalence of body dissatisfaction, which improves at the 6-month follow-up following treatment (66).

With this in mind, it is important to resize the patient’s expectations about weight loss (often overestimated with respect to the real therapeutic goal) through better communication by the healthcare professional or the multidisciplinary team and to pay more attention during the initial phase of treatment.

Moreover, this review revealed that most of the included articles showed that CBT led to significant improvements in psychological variables and BED episodes. In 2016, Calugi et al. (54) concluded that although the BED group maintained higher psychological impairment than the group without BED at 6 months, more than half of the BED patients were no longer diagnosable at 5 years follow-up.

In studies where CBT was used as the only approach, the dropout rate ranged between 10 and 62%. Not all studies considered the same follow-up period, and in studies with longer follow-up periods, the dispersion increased exponentially. Nevertheless, in the study by Brambilla et al. (39) where CBT was used in both the intervention and control groups, the dropout rate was low (16%). Since CBT was the only therapy common to all three interventions, it is probably effective in preventing dropout independently of the results obtained, as shown by the authors.

Furthermore, there were several studies where CBT was used in the intervention group, and the dropout rate was higher than or equal to the control group. In the study by Mefferd et al. (34) dropouts (10.6%) were assigned to the intervention group. However, considering that the control group consisted of a wait-list, it is difficult to conclude the effectiveness of CBT. In a study by Stahre et al. (36) there were no significant differences between the two groups. Although, the percentage of completers was very high in both cases (87% in the intervention group with CBT and 80% in the control group). Donini et al. (41), instead, showed higher treatment duration in Nutritional Psycho-Physical Reconditioning (NPPR) and a significantly lower dropout rate (5.5% vs. 54.4% in standard diet intervention). In addition, weight loss and fat mass reduction were higher in NPPR. The authors of this study hypothesized that the low dropout rate could be ascribed to the multidisciplinary and cognitive-behavioral approach, which provides effective tools to address barriers that usually hinder compliance (e.g., establishing acceptable goals) and increase patients’ motivation to adhere to the procedure. They also affirmed that the improvement in anxiety and depression in the NPPR group allowed one to maintain an adequate lifestyle and sustain the achieved results.

The data from this review have clinical implications as they could help clinicians identify those at a higher risk of dropping out by investigating specific factors as best as possible. In fact, the importance of motivation in the failure of weight loss treatment makes the assessment of motivation a core procedure for all patients with obesity and overweight, both before and during treatment. It has been recently suggested that the importance of motivation in the failure of weight loss programs makes the assessment of motivation a core procedure for all patients living with obesity, both before and during treatment. Armstrong et al. suggested that a motivational interview (a directive, patient-centered counseling approach focused on exploring and resolving ambivalence) appears to enhance weight loss in people with overweight or obesity (67). Moreover, the motivational interview could be used as a separate intervention throughout the course of treatment, when the motivation of obese patients decreases (68). Furthermore, the dissatisfaction with the initial results of the treatment association with dropouts indicates that intensive treatment in the first part of the program might be useful. For example, increasing the number of sessions, offering them closer together, or even offering intermediate telephone contacts could be a potentially effective way to increase the initial weight loss rate and consequently reduce the dropout rate.

This study has several limitations. Most of the studies included, especially those added manually, had an observational and non-randomized design, which resulted in a high risk of bias, as shown in Figure 3. This data could probably be because of the decision to add several articles published by the same research group (28, 29, 33, 40, 50, 53, 54, 56, 60, 61), notwithstanding that this is a leading expert team and permitted a better understanding of the advantages and considerations of CBT. Despite the high risk of bias, the observational design allows clinicians to analyze and comprehend the complex phenomenon of obesity and evaluate numerous variables. Another limitation may be derived from the search strategy because the acronym CBT can be used for both “cognitive behavioural therapy” and “cognitive behavioural treatment” or “cognitive behavioural theory.” Moreover, most of the time, these terms are also reported with “-” divisors. Furthermore, it’s not possible to define the exact approach used in the selected studies; in particular, if the authors used generic forms of CBT or specific form of CBT for obesity management. Indeed, a specific form of CBT, called personalized CBT for obesity (CBT-OB), has been developed and widely studied in recent decades. The main goals of CBT-OB are to help patients to (i) reach, accept and maintain a healthy amount of weight loss (i.e., 5–10% of their starting body weight); (ii) adopt and maintain a lifestyle conducive to weight control; and (iii) develop a stable “weight-control mind-set.” Specific integrations enable the treatment to be personalized, and help patients address with specific strategies and procedures the processes that could be, respectively, associated with drop-out, the amount of weight lost, and maintaining a lower weight in the long term treatment. CBT-OB therapists adopt a therapeutic style designed to develop and nurture a collaborative working relationship (the therapist and patient(s) work together as a team) (69, 70). Given the prevalence of long lasting eating disorders (ED) and their association with high attrition from weight management programs, the search strategy could have included specific terms and amplified the results, this could be addressed in future studies. The strength of the search lies in being systemic and in including all articles concerning CBT in treating obesity, regardless of other correlated pathologies.

Future research with well-designed randomized clinical trials involving different behavioral approaches could focus on answering how it could affect the adherence to the treatment and prevent the dropping out of adults living with overweight and obesity.

## Conclusion

High attrition is a common problem in weight loss interventions and seriously affects weight loss management and frustration. The purpose of this current systematic review was to determine the predictive factors of dropout in treatment of people with overweight and obesity. The main predictive factors are younger age and baseline BMI/weight; and the common motivations of dropping out are dissatisfaction with the result or treatment, personal issues and health problems. Moreover, this review provides additional evidence with respect to CBT leading to significant improvements in psychological variables. These findings have important clinical implications as they could help clinicians identify those at a higher risk of dropping out, support them during the intervention, or find more suitable intervention options.

However, this review highlights the need for more rigorous and well-designed clinical trials to provide more definitive evidence. Ultimately, a deeper understanding of the comparative effectiveness of these treatment strategies is of great value to patients, clinicians, and healthcare policymakers.

## Data availability statement

The original contributions presented in the study are included in the article/supplementary material, further inquiries can be directed to the corresponding author.

## Author contributions

CF and FM: conceptualization. LN, CF, and AT: methodology. LN, MG, CF, and FM: investigation and writing – original draft preparation. LN, CF, FM, MG, SF, and AT: data curation and writing—review and editing. AT and CF: supervision. All authors have read and agreed to the present version of the manuscript.
